# A 7‐year‐old female with hypotonia and scoliosis

**DOI:** 10.1111/bpa.13076

**Published:** 2022-06-05

**Authors:** Yoshihiko Saito, Shimpei Baba, Hirofumi Komaki, Ichizo Nishino

**Affiliations:** ^1^ Department of Neuromuscular Research National Institute of Neuroscience, National Centre of Neurology and Psychiatry Tokyo Japan; ^2^ Department of Clinical Genome Analysis Medical Genome Center, National Center of Neurology and Psychiatry Tokyo Japan; ^3^ Department of Child Neurology National Institute of Neuroscience, National Center of Neurology and Psychiatry Tokyo Japan; ^4^ Translational Medical Center National Center of Neurology and Psychiatry Tokyo Japan

**Keywords:** immunohistochemistry, muscle pathology, myopathy, skeletal muscle imaging

## 1 CASE PRESENTATION

**FIGURE 1:**
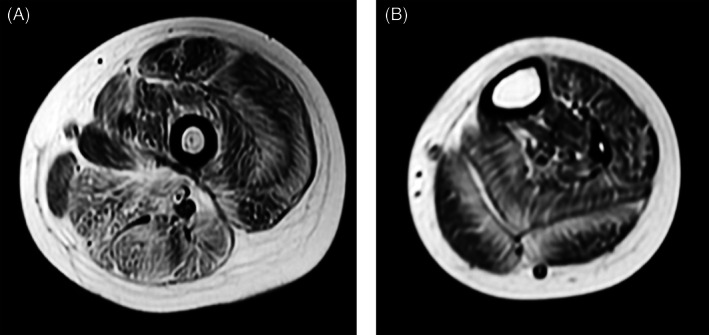
(A, B) High intensity observed on T1‐weighted magnetic resonance imaging in the peripheral area of the vastus lateralis (white arrow), soleus, and gastrocnemius muscles (white arrow head), in addition to the central area of the rectus femoris muscle (black arrow head)

The patient was referred to our hospital at age 7. She was delivered normally at 40 weeks of gestation and had no family history of neuromuscular disease. No abnormal findings were reported during regular antenatal care. Her height and weight at birth were 48 cm and 2696 g, respectively. The patient was found to have scoliosis and hip dislocation at the age of 2 months. She held her head up at 4 months and became ambulatory at 3 years. Though she had contractures of the proximal joints, hyperlaxity of the distal joints was not observed. There was no delay in intellectual development. At presentation, her height and weight were 86.5 cm (−7.0 SD) and 10 kg (−3.2 SD), respectively. Blood chemistry testing revealed that the aspartate aminotransferase, alanine aminotransferase, lactate dehydrogenase, and creatine kinase levels were within the normal range. No abnormalities were observed in nerve conduction studies. On muscle magnetic resonance imaging (MRI) of the lower extremities, high intensity on T1‐weighted imaging was observed in the peripheral area of the vastus lateralis (white arrow), soleus, and gastrocnemius muscles (white arrowhead), in addition to the central area of the rectus femoris muscle (black arrowhead) (Box 1; Figure 1A,B).

BOX 1 **Slide scan**

Access the whole slide scan at http://image.upmc.edu:8080/NeuroPathology/BPA/BPA‐21‐10‐255.svs/view.apml

## 2 FINDINGS

**FIGURE 2:**
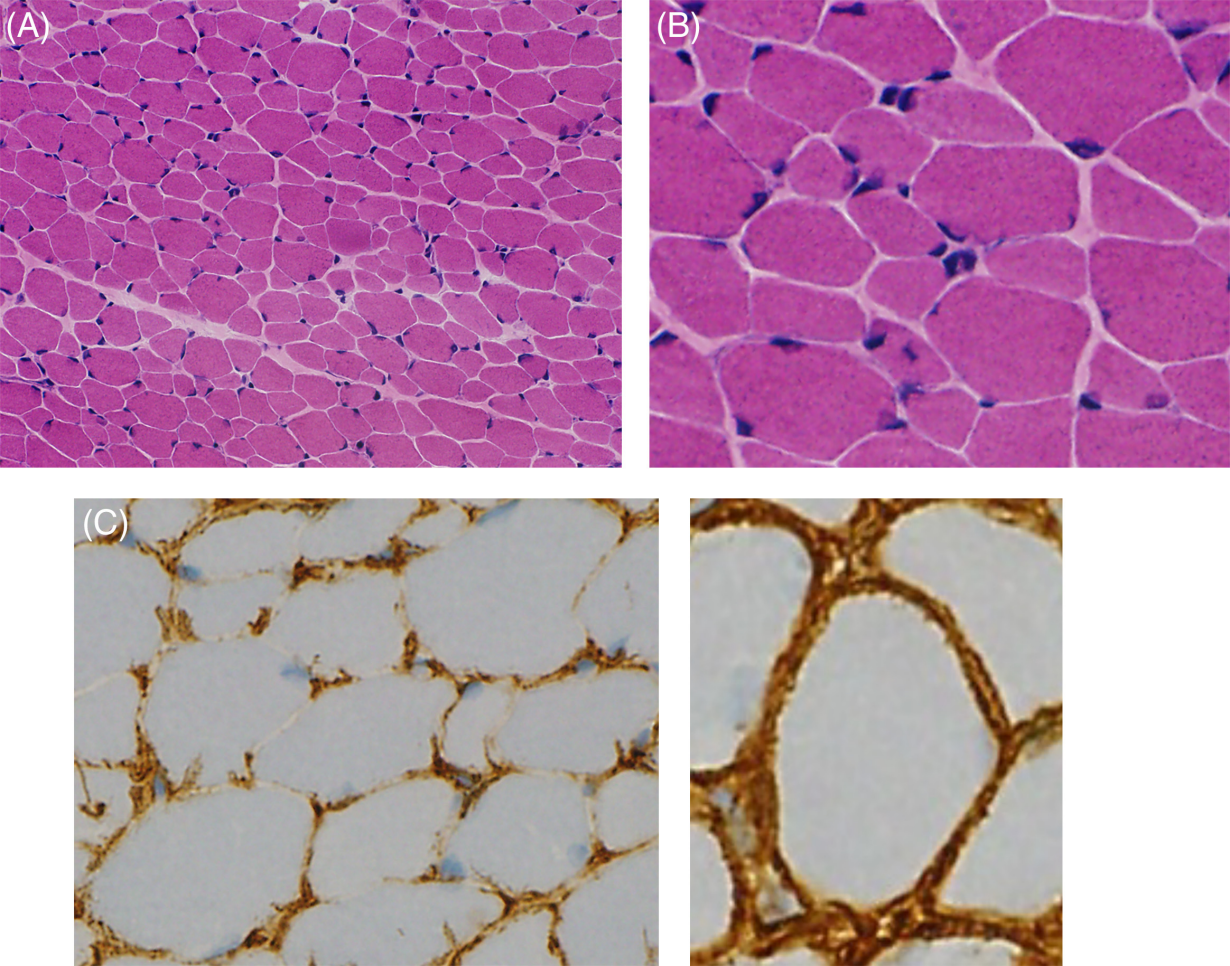
(A and B) hematoxylin and eosin staining indicated a marked variation in fiber size. Fibers with internalized nuclei are scattered. Mild endomysial and perimysial fibrosis was observed. (C and control) immunohistochemistry images showing collagen VI deficiency in the sarcolemma and presence in the interstitium

A muscle biopsy was performed using a specimen from the left biceps brachii. A battery of histochemistry including hematoxylin and eosin (H&E), modified Gomori trichrome, NADH‐tetrazolium reductase, and myosin ATPase were performed. There was a marked variation in fiber size, ranging from 10 to 35 μm in diameter. Fibers with internalized nuclei were scattered although no necrotic or regenerating fibers were observed. Mild endomysial and perimysial fibrosis were observed (Figure 2A,B; [H&E] staining). Mononuclear cell infiltration was not observed. Intermyofibrillar networks were mildly disorganized in scattered fibers, occasionally showing multiminicore‐like structures. Type 2C fibers were scattered. On immunohistochemistry, collagen VI was found to be deficient in the sarcolemma but present in the interstitium (Figure 2C and control; collagen VI) while all other muscular dystrophy‐related proteins were normally expressed, including dystrophin, sarcoglycans, alpha‐dystroglycan, dysferlin, and caveolin 3. Moreover, and there was no expression of HLA‐ABC, HLA‐DR and myxovirus resistant protein A.

## 3 DIAGNOSIS

Collagen VI‐related myopathy/Ullrich congenital muscular dystrophy (UCMD). Genetic analysis of the *COL6A2* gene using genomic DNA revealed a previously reported heterozygous mutation, c.902G>A (p.G301D) in exon 8 confirming the diagnosis.

## 4 DISCUSSION

Collagen VI‐related myopathies are known to encompass a clinical continuum with UCMD and Bethlem myopathy (BM) at each end of the spectrum. Collagen VI‐related myopathies are caused by mutations in the triple helical domain (THD) (2). Collagen VI is an extracellular matrix protein composed of α1, α2, and α3 chains encoded by *the COL6A1, COL6A2*, and *COL6A3* genes, respectively. Each of the three collagen VI chains has a THD, consisting of 335–336 amino acids with repeating Gly‐Xaa‐Yaa amino acids in the central region (1, 2), which is flanked by amino‐ and carboxyl‐globular domains. The three chains are folded, through the interaction at the THD, into collagen VI monomers that are further assembled into dimers and tetramers prior to secretion from cells. In the extracellular space, tetramers are associated in an end‐to‐end manner into the characteristic double‐beaded collagen VI microfibrils. Therefore, mutations affecting multiple assembly steps can have strong dominant negative effects, resulting in the absence of collagen VI microfibrils (2).

The MRI findings of collagen VI‐related myopathy seem to be highly specific to the disease (1, 2). On axial images through the thigh, the periphery of the vastus lateralis was markedly infiltrated by fat, while the central region was spared. Furthermore, the central part of the rectus femoris was markedly infiltrated by fat while the periphery was spared. These findings are called “tigroid” and “target” signs (1, 2), respectively, with sensitivities of 63.6% and 90.9% and specificities of 97.3% and 96.9% for the diagnosis of collagen VI‐related myopathies (1). Similarly, at the mid‐calf, the periphery of the soleus and gastrocnemius muscles are also markedly infiltrated by fat (2). These areas causing localized fat replacement include muscle‐tendon junctions, which may reflect the fragility of the muscle‐tendon junctions in this disease. The severity of the involvement on MRI in patients with UCMD and BM is not significantly different; however, it is more closely associated with the severity of clinical involvement than with age.

Normally, on immunohistochemistry of the skeletal muscle, collagen VI is prominently observed in the sarcolemma and partly in the interstitium (2, 3). In contrast, in the majority (85%) of the patients with UCMD, collagen VI was almost completely deficient from the sarcolemma, while it was present in the interstitium, known as sarcolemma‐specific collagen VI deficiency (SSCD). Additionally, a minority (15%) of patients showed complete deficiency (CD) (3). SSCD is caused by a sporadic dominant mutation in the regions located at the N‐terminal side of the cysteine residue within the THD of either *COL6A1, COL6A2*, or *COL6A3*, while CD is caused by a recessive mutation of any of the three *COL6* genes. Although the remarkable gait acquisition, hyper relaxation of the distal joint, and arthrogryposis are consistent with UCMD, it is possible that it is an intermediate form. However, this variant has been previously reported as a UCMD form and is considered to be consistent with UCMD along with clinical manifestations. If the detected variant is in the triple helix domain, which is the hotspot of the disease, and was previously reported for collagen VI‐related myopathy, diagnosis can be made based on clinical information and genetic analysis without examining muscle pathology.

Our case was a typical case of UCMD with a reported variant in the triple‐helical domain, the major site of the disease. The assessment of a sarcolemmal collagen VI deficiency on muscle pathology can provide useful information leading to a confident diagnosis of collagen VI‐related myopathy especially when the pathogenic interpretation of the genetic variant is difficult.

## AUTHOR CONTRIBUTIONS

Yoshihiko Saito: drafting the manuscript, acquisition of data and interpretation of pathological results. Shimpei Baba and Hirofumi Komaki: collecting clinical data. Ichizo Nishino: study design and supervision of study. All authors read and approved the final manuscript.

## CONFLICT OF INTEREST

Ichizo Nishino received grants from JSPS KAKENHI Grant Numbers (21K06961, 20K16589, JP21K15689, 20H03592, 19K11459, 20K07911); Intramural Research Grant (2‐5, 2‐6, 2‐4, 3‐9) for Neurological and Psychiatric Disorders of NCNP; AMED under Grant Numbers, JP21ek0109490h0002, JP21ek0410074s1302, JP21ek0109493s0502; and Science Research Grants from the Ministry of Health, Labour and Welfare (JPMH20FC1036, JPMH21FC1006). Yoshihiko Saito, Shimpei Baba, and Hirofumi Komaki report no disclosures.

## ETHICS STATEMENT

All clinical information and materials used in this study were obtained for diagnostic purposes with written informed consent. This study was approved by the ethics committee of the National Center of Neurology and Psychiatry in Japan. This patient's guardians provided written informed consent for the publication of the case details.

## Data Availability

Data sharing is not applicable to this article as no new data were created or analyzed in this study.
